# Biological Markers of High-Risk Childhood Acute Lymphoblastic Leukemia

**DOI:** 10.3390/cancers16050858

**Published:** 2024-02-21

**Authors:** Jiasen He, Faryal Munir, Samanta Catueno, Jeremy S. Connors, Amber Gibson, Lindsay Robusto, David McCall, Cesar Nunez, Michael Roth, Priti Tewari, Sofia Garces, Branko Cuglievan, Miriam B. Garcia

**Affiliations:** 1 Department of Pediatrics, The University of Texas MD Anderson Cancer Center, Houston, TX 77030, USAdmccall1@mdanderson.org (D.M.);; 2 Department of Hematopathology, The University of Texas MD Anderson Cancer Center, Houston, TX 77030, USA

**Keywords:** pediatric, high-risk acute lymphoblastic leukemia, childhood cancer

## Abstract

**Simple Summary:**

Childhood acute lymphoblastic leukemia (ALL) has seen significant advances in treatment, yet children classified as high-risk still face challenging outcomes. Traditionally, the severity of ALL was assessed using basic clinical information at diagnosis, but now a deeper understanding of specific biological markers—such as molecular profiles, genetic variations, and immune system characteristics—has become crucial. These markers are not just keys to understanding the disease’s mechanisms, but also indicators of how it may progress and respond to treatment. For instance, the development of drugs like tyrosine kinase inhibitors can be used to target high-risk leukemia with certain genetic mutations. By focusing on the intricacies of high-risk childhood ALL, research is paving the way for more personalized and precise treatments, offering hope for better management of this complex disease.

**Abstract:**

Childhood acute lymphoblastic leukemia (ALL) has witnessed substantial improvements in prognosis; however, a subset of patients classified as high-risk continues to face higher rates of relapse and increased mortality. While the National Cancer Institute (NCI) criteria have traditionally guided risk stratification based on initial clinical information, recent advances highlight the pivotal role of biological markers in shaping the prognosis of childhood ALL. This review delves into the emerging understanding of high-risk childhood ALL, focusing on molecular, cytogenetic, and immunophenotypic markers. These markers not only contribute to unraveling the underlying mechanisms of the disease, but also shed light on specific clinical patterns that dictate prognosis. The paradigm shift in treatment strategies, exemplified by the success of tyrosine kinase inhibitors in Philadelphia chromosome-positive leukemia, underscores the importance of recognizing and targeting precise risk factors. Through a comprehensive exploration of high-risk childhood ALL characteristics, this review aims to enhance our comprehension of the disease, offering insights into its molecular landscape and clinical intricacies in the hope of contributing to future targeted and tailored therapies.

## 1. Introduction

Acute lymphoblastic leukemia (ALL), a hematopoietic malignancy of B or T lymphoblasts, is the most common pediatric malignancy. Of the approximately 5690 new ALL cases in 2021 in the USA, 53.5% were diagnosed in patients younger than 20 years [1,2]. B-cell ALL (B-ALL), which accounts for 85–90% of pediatric ALL and around 75% of adult ALL, is more common than T-cell ALL (T-ALL) [3].

Aggressive combinations of traditional chemotherapy have dramatically improved the overall survival (OS) of newly diagnosed pediatric ALL patients, whose 5-year OS rate has increased from 57% in 1975 to more than 90% today [4]. However, patients with refractory or relapsed ALL have a much poorer prognosis. Primary refractory ALL, which is usually defined by induction therapy failure, has an overall risk of only 2.4–3.8% but a much worse prognosis [5,6]; patients with refractory disease frequently have high-risk features [6]. Around 15–20% of ALL patients, including those without refractory disease, have disease relapse, and these patients’ 5-year OS rate is only 36% [7]. Thus, it is critical to identify ALL patients with high-risk features and pair these patients with the most effective treatments accordingly.

**Figure 1:**
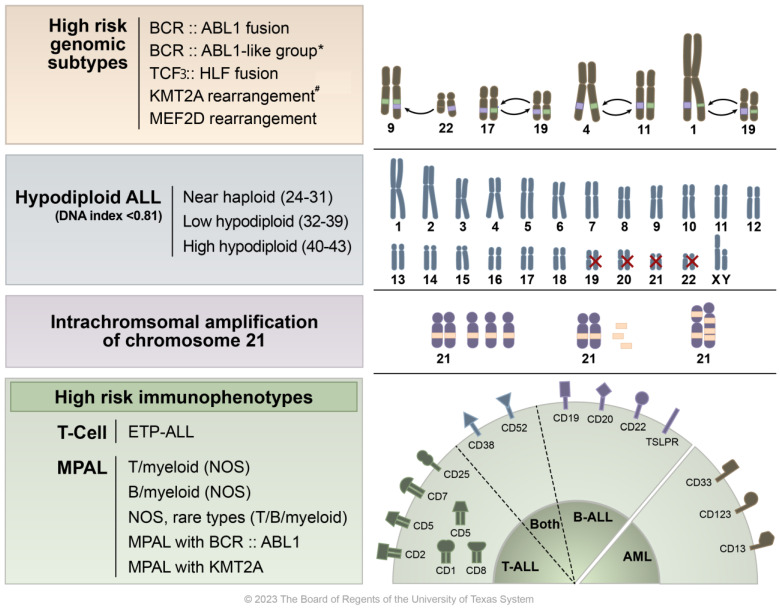
Overview of high-risk ALL molecular, cytogenetic, and immunophenotypic findings. This figure presents a graphical representation of the various high-risk characteristics found in acute lymphoblastic leukemia (ALL). At the top, high risk genomic features are listed alongside illustrated examples of chromosomal translocations/gene fusions/rearrangements, specifically *BCR::ABL1* t(9;22), *TCF3::HLF* fusion t(17;19), *KMT2A* rearrangement t(4;11), and *MEF2D* rearrangement t(1;19). * Although BCR::ABL1-like ALL shares genetic characteristics with Ph+ ALL, it lacks the *BCR::ABL1* translocation abnormality. The second row details subtypes of hypodiploid ALL, including one that features the loss of chromosomes 19, 20, 21, and 22. The third row highlights cases where additional copies of a chromosome 21 region, which includes the *RUNX1* gene, are present in excess (five or more copies per cell). The bottom row categorizes various high-risk immunophenotypes including early T-cell precursor ALL (ETP-ALL) and mixed phenotype acute leukemia (MPAL), along with their corresponding CD markers. ^#^ *KMT2A*/11q23 has over 100 fusion partners identified, with the most common being t(4;11)—AFF1, t(9;11)—MLLT1, and t(11;19)—MLLT3 (Meyer et al.) [12].

In ALL patients, “risk-adapted therapy”, which is based on clinical and biological risk factors, can guide treatment intensity to maximize the chance of cure and minimize toxicity [8,9]. The appropriate risk-adapted therapy for individual ALL patients is determined using risk stratification systems. The traditional risk stratification system is based on the National Cancer Institute (NCI) criteria, which was adopted by the Pediatric Oncology Group and Children’s Cancer Group in 1993. The NCI criteria include age, initial white blood cell (WBC) count, and extramedullary disease status at diagnosis [10]. Since the Pediatric Oncology Group and Children’s Cancer Group merged to form the Children’s Oncology Group (COG), risk stratification systems with additional factors have emerged [11]. In addition to the NCI criteria, risk stratification systems now consider early treatment response, which is usually determined by minimal residue disease (MRD) status after induction and consolidation therapy, as well as immunophenotype and sentinel cytogenetic findings or molecular mutations, which is summarized in Figure 1 [9,11].

Identifying and comprehending the high-risk features of ALL is crucial to the development of targeted interventions. In this article, we delineate the immunophenotypes, chromosomal lesions, and genetic mutations associated with high-risk ALL and focus on HR features which currently have either clinical trials or strong preclinical data to inform trials in the near future. Survival data and available targeted therapies for patients with high-risk ALL are summarized in Table 1.

## 2. High-Risk Features

### 2.1. High-Risk Molecular Genomic Subtypes

#### 2.1.1. B-Lymphoblastic Leukemia/Lymphoma with BCR::ABL1 Fusion

The reciprocal translocation t(9;22) leads to the Philadelphia chromosome abnormality, which causes 2–5% of pediatric ALLs [38]. The Philadelphia chromosome, whose incidence increases as age advances, is the most common chromosomal abnormality in adult ALL, with an overall incidence of 20–25% [38,39,40,41]. Philadelphia chromosome-positive ALL (Ph+ ALL) is historically associated with worse outcomes, with long-term survival rates of 10–20% [41,42,43,44]. Before the availability of tyrosine kinase inhibitors (TKIs), hematopoietic stem cell transplantation (HSCT) provided a cure in only 50–60% of patients during the first remission [42,45,46].

The *BCR::ABL1* fusion oncoprotein that results from the reciprocal translocation t(9;22) has intrinsic tyrosine kinase activity [47,48,49]. *BCR::ABL1* fusion leads to the upregulation of several cell cycle signaling pathways, including RAS/RAF/MEK/ERK, PI3K/AKT/mTOR, and JAK/STAT, and is associated with the activation of other tyrosine kinases such as SRC family members (e.g., LYN, HCK) and MYC [49,50,51]. The aberrant expression of the *BCR::ABL1* fusion oncoprotein in lymphohematopoietic cells results in dysregulated cell proliferation and reduced apoptosis through deregulated tyrosine kinase activity, making the protein an excellent molecular therapeutic target [52].

In chronic myeloid leukemia (CML), the BCR gene breakpoint typically occurs in the major breakpoint cluster region (M-BCR), leading to the production of a 210 kD BCR-ABL1 fusion protein (p210), whereas in Ph+ ALL, the breakpoint may be in either the M-BCR or the minor BCR (m-BCR), resulting in a 190 kD fusion protein (p190) [53,54]. The clinical presentation of CML in lymphoid blast crisis (BC) closely resembles Ph+ ALL, posing diagnostic challenges, particularly when M-BCR rearrangements are present and associated with the p210 protein, which is characteristic of CML [53,55]. This distinction is crucial for treatment decisions, as Ph+ ALL typically warrants chemotherapy combined with a tyrosine kinase inhibitor, while CML presenting in or progressing to BC often necessitates allogeneic stem cell transplantation, highlighting the importance of accurate leukemia subtyping for optimal therapeutic strategies [55].

The introduction of TKIs in the treatment of Ph+ ALL brought breakthrough improvements in outcomes and, hence, became part of standard-of-care frontline therapy. The COG AALL0031 study reported that the combination of the TKI imatinib with chemotherapy doubled the 5-year disease-free survival (DFS) rate of children with very high-risk Ph+ ALL to 70% [13]. A second-generation TKI, dasatinib, has a potency more than 300-fold that of imatinib and can permeate the blood–brain barrier, making it useful in the treatment of ALL patients with central nervous system disease; however, it does not completely prevent central nervous system relapse [22,56,57]. The COG AALL0622 study of patients with Ph+ ALL in which dasatinib was started on day 15 of induction therapy at a dose of 60 mg/m^2^/day, showed that the treatment, even in the absence of cranial irradiation, had results similar to those observed in COG AALL0031. COG AALL0622 also supported restricting HSCT to only slow responders [57]. A slightly higher dose of dasatinib (80 mg/m^2^/day) was investigated by the Chinese Children’s Cancer Group, who also found significant improvements in event-free survival (EFS) and OS, as well as fewer relapses compared with those who received imatinib [22,58].

An analysis of long-term follow up data from the EsPhALL2004 study, in which pediatric Ph+ ALL patients were treated with imatinib, yielded results similar to those observed in COG AALL0031 [13] and COG AALL0622 [57], thus emphasizing the need for early TKI exposure and de-emphasizing the need for HSCT in the future trials. This follow-up study also concluded that a WBC count of at least 100 × 10/L at the time of Ph+ ALL diagnosis predicted a worse prognosis [59].

The ideal TKI treatment duration has not been established conclusively; indeed, unless intolerable toxicity occurs, TKI treatment can last indefinitely [60,61,62]. In patients with Ph+ ALL, the ultimate goal of therapy is a sustained complete molecular response, which may be an independent prognostic factor for increased OS and may preclude the need for HSCT [41,63]. Additional studies to determine the duration are needed.

#### 2.1.2. B-Lymphoblastic Leukemia/Lymphoma with BCR::ABL1-like Features

Philadelphia chromosome-like ALL (Ph-like ALL) is a recently discovered aggressive entity that shares genetic characteristics with Ph+ ALL, but lacks the BCR::ABL1 translocation abnormality [41,64,65]. Ph-like ALL is thrice as common as Ph+ ALL, representing about 10% of pediatric ALL, 15–25% of adolescent and young adult ALL, and 20–27% of adult ALL [15,16]. Among young adults of Hispanic descent, Ph-like ALL has a high prevalence (>50%) [15,66], which may be partially associated with the ethnic group’s high rate of rearrangements of the cytokine receptor-like factor 2 gene *CRLF2* [15,67]. Ph-like ALL has adverse clinical traits and a dismal prognosis, with an estimated survival rate of less than 30% [15]. Furthermore, Ph-like ALL also has an increased association with Down syndrome [68,69].

Ph-like ALL has a complicated genomic landscape; the findings of genome and transcriptome sequencing studies suggest a variety of genetic changes that dysregulate various classes of cytokine receptors and tyrosine kinases [70]. Like those with Ph+ ALL, most patients with Ph-like ALL (70%) have hallmark *IKZF1* alterations [66,69,71,72], which are associated with high rates of induction therapy failure and a high risk of relapse [57,73].

It is worth noting that while the Ph-like gene expression signature holds diagnostic value, it has not yet been identified as a therapeutic target. For example, strategies for targeting therapy for *IKZF1* deletion are still not well-defined [68]. However, it is important to consider sentinel molecular lesions that are instrumental in driving leukemogenesis as potential targets. These alterations can be broadly categorized into major subclasses, such as ABL-class fusions, lesions that activate JAK/STAT signaling, and others that affect different signaling pathways.

The kinases altered in ABL-class fusions that phenocopy *BCR-ABL1* include platelet-derived growth factor receptor alpha (PDGFRA) and beta (PDGFRB), colony stimulating factor 1 receptor (CSF1R), and ABL1 and ABL2, which provide targets for ABL inhibitors [74]. TKIs have shown efficacy against Ph+ ALL, as well as Ph-like ALL and T-ALL with ABL1-class fusions [75,76,77,78]. The alteration of CRLF2, JAK2, and EPOR can activate JAK/STAT signaling; thus, JAK2 inhibitors can potentially be used in patients with these alterations [68]. More than half of patients with Ph-like ALL have *CRLF2* rearrangements, and of those with such rearrangements, 50% have concurrent activating mutations of Janus kinases (JAK1, JAK2, and JAK3) [41,71]. Other cytokine receptor alterations involve PI3K, mTOR, and the JAK/STAT pathways. Mutations in JAK2 and EPOR are present in about 7% and 5% of cases, respectively, and are associated with worse outcomes [15,69]. Additionally, 4–10% of Ph-like ALL have mutations in RAS pathway members, including *KRAS*, *NRAS*, *NF1*, *PTPN11*, and *CBL1* [69].

According to the COG AALL0331 study, Ph-like ALL is less prevalent in children with NCI standard-risk ALL than in those with high-risk ALL [79]. In one phase 3 randomized controlled trial in patients with high-risk ALL (COG AALL0232), the 5-year EFS rate for patients with Ph-like ALL (63%) was lower than that for patients with non-Ph-like ALL (86%) [80,81]. To this end, a phase 2 trial (COG AALL1521) is evaluating the efficacy and dosage of ruxolitinib combined with chemotherapy in patients with Ph-like ALL with *CRLF2* rearrangements and/or additional JAK-STAT pathway abnormalities [15]. In Europe, the AIEOP-BFM ALL and ALLTogether study groups are also investigating the use of novel or targeted therapies combined with chemotherapy in patients with Ph-like ALL [16].

Additional clinical trials are investigating the role of combining dasatinib with chemotherapy in patients with relapsed or newly diagnosed ABL-mutated Ph-like ALL. A phase I/II study (NCT02420717) initiated at MD Anderson assessed the safety and efficacy profile of dasatinib with hyper-CVAD (cyclophosphamide, vincristine, doxorubicin, dexamethasone) in adolescents and young adults with relapsed or recurrent Ph-like ALL with ABL-class fusions. Although preliminary data demonstrated the safety of this combination therapy, the study was closed, owing to low accrual, and the combination did not move to phase II [82]. The potential efficacy of dasatinib combination therapy in children and adolescents and young adults with de novo ALL with ABL-class mutations and Ph-like ALL is being investigated in the phase 3 COG AALL1131 trial (NCT01406756) and the SJCRH Total XVII trial (NCT03117751). Although initial results indicated TKIs to have beneficial effects, the studies’ final results have not yet been published. There are ongoing efforts to add immunotherapy agents to upfront regimens [83].

#### 2.1.3. B-Lymphoblastic Leukemia/Lymphoma with *KMT2A* Rearrangement

The lysine methyltransferase 2A gene *KMT2A* (also known as *MLL*), located on chromosome 11q23, can be rearranged with different gene loci and can occur in acute leukemias of lymphoid or myeloid origin [18]. *KMT2A* gene rearrangements occur in 10–15% of adult patients with B-ALL, but only 5% of children and young adults with ALL [84]. They are also found in about 70% of infants with ALL and are believed to be acquired in utero [18]. In infant ALL, *KMT2A* gene rearrangements are linked to poor prognosis, particularly in infants diagnosed before the age of 6 months, present with a WBC count of at least 300 × 10^9^/L, or have a poor response to induction therapy with steroids [85]. *KMT2A*-rearranged ALL is a high-risk subgroup with dismal treatment responses and a long-term survival rate of less than 60% [84,86]. Furthermore, therapy responses vary based on specific translocations. The Ponte-di-Legno Childhood ALL Working Group’s recent retrospective study of 629 patients with 11q23/*KMT2A*-rearranged ALL reported a 5-year EFS rate of 69.1 ± 1.9% for the entire cohort, but a range of rates for patient subgroups. For instance, the 5-year EFS rate for patients with t(9;11)-positive T-ALL (*n* = 9) was 41.7 ± 17.3%, whereas that for patients with t(4;11)-positive B-ALL (*n* = 266) was 64.8 ± 3.0% and that for patients with t(11;19)-positive T-ALL (*n* = 34) was 91.2 ± 4.9% [87].

Two international randomized studies of infant patients with *KMT2A*-rearranged ALL found no appreciable differences in outcomes between standard and intensified chemotherapy (the Interfant-99 study) or between myeloid- and lymphoid-type consolidation therapy (the Interfant-06 study) [17,88]. Recently, Stutterheim et al. looked at the clinical implications of MRD in infants with *KMT2A*-rearranged ALL treated on the Interfant-06 protocol. The study demonstrated an improved DFS based on stratification of therapy according to MRD at the end of induction. Infants with high MRD levels at the end of induction therapy benefited more from acute myeloid leukemia (AML)-like consolidation therapy (6-year DFS, 45.9%) than from ALL-like consolidation therapy (23.2%), whereas those with low MRD levels at the end of induction therapy may respond better to ALL-like consolidation regimens (6-year DFS, 78.2%) than to AML-like regimens (45.0%); patients with MRD at end of consolidation therapy continued to have grim outcomes. These results will pave the way for more treatment interventions in the next Interfant protocol [85].

*KMT2A* rearrangement encourages the formation of a unique multi-protein complex that comprises DOT1L, BRD4, and menin and, thus, is a potential molecular target for DOT1L, bromodomain, menin, and BCL2 inhibitors [22,89]. The immunotherapy agent blinatumomab and chimeric antigen receptor (CAR) T-cell therapy are also under consideration [18]. One recent study showed that blinatumomab in combination with the chemotherapy regimen used in the Interfant-06 trial had notable safety and remarkable efficacy in infants with newly diagnosed *KMT2A*-rearranged ALL; these outcomes surpassed those of historical controls in the Interfant-06 trial [90]. Menin inhibitors are also emerging as promising therapeutic agents against *KMT2A*-rearranged leukemias, with an overall response rate of 55% and a favorable adverse event profile from the Augment-101 trial; however, this should be viewed with caution given the low number of pediatric patients and that infants were not included in this trial [19].

#### 2.1.4. B-Lymphoblastic Leukemia with MEF2D Rearrangement

The myocyte-specific enhancer factor 2D gene *MEF2D*, located on 1q22, belongs to the MEF2 family, which encodes a group of transcription factors that control muscle and neuronal cell differentiation and development and are regulated by class II histone deacetylases (HDACs) [91,92]. *MEF2D* rearrangement was recently identified in a subgroup of B-ALL patients presenting with high-risk features [21,93,94]. The fusion partners of *MEF2D* include its most common partner, *BCL9* (located on 1q21), as well as *CSF1R* (5q32), *DAZAP1* (19p13.3), *HNRNPUL1* (19q13.2), *SS18* (18q11.2), and *FOXJ2* (12p13.31) [21,94]. These *MEF2D* fusions are thought to enhance *MEF2D* transcription activity, which leads to the development of high-risk ALL [21,95]. Interestingly, *KMT2A*-rearranged AML has high *MEF2D* expression, which might play a critical role in leukemia development [96].

The true incidence of *MEF2D* fusions is unknown because they are not included in routine screening; however, the fusions have been reported in 2–4% of pediatric and 7% of adult precursor B-ALL [21,93,95,97,98]. *MEF2D* fusion-positive ALL has a unique immunophenotype; it has weak or no expression of CD10, aberrant expression of CD5, and frequent expression of CD38 and cytoplasmic μ chain [21,98]. Patients with *MEF2D* fusion-positive ALL have a median age of 9 years at diagnosis and have elevated WBCs at presentation [98]. Suzuki et al. reported that, among four ALL patients with initial relapse, those with *MEF2D::BCL9* fusion were more likely to have had induction therapy failure and early relapse [93]. Ohki et al., in a study of ALL patients without known major risk-stratifying cytogenetic abnormalities, identified 17 patients with *MEF2D* fusion [98]; among the 15 patients for whom data were available, 8 had relapse and died from the disease [98].

Because the MEF2 transcription factor family is regulated by HDACs, HDAC inhibitors have been explored as therapeutic options and were found to be effective against *MEF2D*-rearranged ALL in vivo [21]. In clinical trials of patients with relapsed/refractory ALL, HDAC inhibitors, including panobinostat and vorinostat, are being investigated alone (NCT00723203) or in combination with chemotherapy (NCT01321346, NCT02518750) or bortezomib (NCT02553460, NCT01312818). These trials will provide more information about the use of HDAC inhibitors in patients with *MEF2D* fusions.

#### 2.1.5. B-Lymphoblastic Leukemia/Lymphoma with TCF3::HLF Fusion

Both the transcription factor 3 gene *TCF3* (also known as *E2A*; locus 19p13) and the hepatic leukemia factor gene *HLF* (locus 17q22) encode transcription factors. The *TCF3::HLF* fusion gene, which results from the translocation t(17;19) (q22;p13), defines *TCF3::HLF* ALL, a rare but aggressive subtype of precursor B-cell ALL [99,100] that accounts for less than 1% of pediatric ALL.

*TCF3::HLF* ALL is usually resistant to conventional chemotherapy and has an extremely poor prognosis even after HSCT [99,100,101]. Patients with *TCF3::HLF* ALL usually present with hypercalcemia and coagulopathy, which can progress to disseminated intravascular coagulation [102]. *BCL2*, a downstream upregulated gene of *TCF3::HLF*, is a druggable target in the disease [103]. Drug response profiling using matched patient-derived xenografts showed *TCF3::HLF* ALL to have striking sensitivity to the BCL2 inhibitor venetoclax [24]. Other options for targeted therapy include PARP inhibitors, since TCF3::HLF expression suppresses homologous recombination repair activity [25]. The PARP inhibitor olaparib has been found to be effective against *TCF3::HLF* ALL both in vitro and in vivo [25]. Because *TCF3::HLF* ALL has high and homogeneous CD19 expression, blinatumomab followed by HSCT was able to induce durable remissions in four of nine patients [26].

### 2.2. High-Risk Cytogenetic Features

#### 2.2.1. Hypodiploid ALL

Hypodiploid ALL, which is identified by the presence of less than 44 chromosomes or a DNA index (the ratio of the amount of DNA in a leukemia sample to the amount of DNA in normal peripheral blood mononuclear cells) of less than 0.81, can be subclassified as near-haploid ALL (24–31 chromosomes), low-hypodiploid ALL (32–39 chromosomes), or high-hypodiploid ALL (40–43 chromosomes). Hypodiploid ALL accounts for 1–2% of pediatric ALL. Hypodiploidy is a poor prognostic factor [22,27,104]. Patients with near-haploid ALL frequently have mutations involving the RAS and PI3K pathways and deletion of *IKZF3* [22,28]. By contrast, 90% of patients with low-hypodiploid ALL have leukemia cells with *TP53* mutations (about 50% of which are germline mutations) or somatic mutations in *IKZF2* and *RB1*. Hence, germline testing for *TP53* mutations (i.e., testing for Li–Fraumeni syndrome) is recommended for patients with low-hypodiploid ALL [22,105,106]. In some cases, hypodiploid clones are duplicated and appear to be pseudo-hyperdiploid clones; it is critical to confirm whether hypodiploid ALL is present to ensure that the correct risk stratification system is used and known risks associated with the disease are identified [22,107].

The preferred treatment modality for patients with hypodiploid ALL is historically HSCT, but is shifting towards molecular therapies. Two recent multicenter studies demonstrated that HSCT confers no benefit in patients with hypodiploid ALL, particularly those who have no MRD after remission-induction therapy, for whom the EFS rate was approximately 70% [22,27,104]. Patients in whom conventional chemotherapy fails to achieve remission can be considered for salvage treatment with BCL2 inhibitors, PI3K inhibitors, immunotherapy, or CAR T-cell therapy [22,28,29,30].

#### 2.2.2. ALL with Intrachromosomal Amplification of Chromosome 21

ALL with intrachromosomal amplification of chromosome 21 (iAMP21) is a cytogenetic subset of pediatric ALL that was first described in 2003 [22,108,109,110]. It is characterized by the amplification of the *RUNX1* gene (≥5 copies per cell) and duplication of chromosome 21 detected with fluorescence in situ hybridization for the *ETV6::RUNX1* fusion gene [22]. iAMP21 seems to arise through multiple breakage–fusion bridge cycles. Patients with the germline Robertsonian translocation rob(15;21) or a germline ring chromosome 21 r(21) have an increased risk of B-ALL with iAMP21 [108,111]. ALL with iAMP21 is a rare but high-risk disease that accounts for 1–2% of pediatric ALL; it is seen more frequently in slightly older children (median age, 9 years) and is associated with lower WBC counts and a grim prognosis [108]. There have been rare instances of iAMP21 co-occurring with other recognized chromosomal abnormalities, such as high hyperdiploidy, *BCR::ABL1*, or *ETV6::RUNX1* [108,112,113]. Otherwise, iAMP21 is a primary cytogenetic abnormality that remains structurally consistent from initial diagnosis through relapse [114]. Similar chromosome 21 anomalies have been observed in myelodysplastic syndromes and AML, typically in conjunction with complicated karyotypes. In those instances, however, chromosomal regions other than that containing *RUNX1* seem to be involved [115,116]. Other cytogenetic abnormalities observed in ALL with iAMP21 ALL include the gain of chromosome X, the loss or deletion of chromosome 7, the deletion of *ETV6* or *RB1*, and the inactivation of *SH2B2* [22,112,117].

In patients with ALL with iAMP21, conventional standard-risk chemotherapy is associated with a poor response and higher relapse rates [31,118]. Intensive chemotherapy regimens offer only slightly better outcomes, with an EFS rate of about 70% [22]. Hence, trials of novel molecular therapies for ALL with iAMP21 are warranted.

### 2.3. High-Risk Immunophenotypes

#### 2.3.1. Early T-Cell Precursor ALL

Early T-cell precursor ALL (ETP-ALL) was recently recognized as a subset of T-cell leukemias with increased molecular heterogeneity [22,41]. ETP-ALL accounts for 10–15% of T-ALL cases and is characterized by genetic features similar to those of hematopoietic stem cells and the early T-cell development immunophenotype (cytoplasmic CD3^+^, CD1a^−^, CD8^−^, CD5^−/dim^) and by some atypical myeloid antigen positivity [41,119]. Compared with T-ALL, ETP-ALL has a lower frequency of *NOTCH1* mutations and higher frequencies of *FLT3* and *DNMT3A* mutations [120,121,122,123]. ETP-ALL has several genomic features similar to those of T/myeloid MPAL, such as an increased incidence of biallelic WT1 changes and mutations in transcriptional regulators; epigenetic regulators; and signaling pathways, including JAK/STAT, RAS, PI3K/AKT/mTOR, FLT3, and MAPK [121,124,125]. Owing to increased glucocorticoid resistance, the disease has an innately poor response to conventional induction therapy, which contributes to a higher incidence of induction therapy failure and persistent MRD [41,119,120,123,126,127]. However, in the COG AALL0434 study, the long-term survival rate of ETP-ALL patients (91%), despite their high rate of MRD at the end of induction therapy, was similar to that of non-ETP-ALL patients (91.5%) [32]. Hence, until more data become available, the current recommendation is to treat ETP-ALL patients on the same protocol as non-ETP-ALL patients [128]. Further clinical trials are warranted to explore the genetic implications of ETP-ALL biology and optimal therapeutic targets.

#### 2.3.2. Mixed-Phenotype Acute Leukemia

Mixed-phenotype acute leukemia (MPAL), which comprises a heterogenous group of uncommon hematological malignancies (not restricted to a single lineage) that express a combination of antigens, accounts for 2–5% of pediatric acute leukemias [125,129,130,131]. MPAL can switch lineages during treatment, which presents extreme diagnostic and therapeutic challenges, owing to a lack of consensus regarding treatment regimens [129,131].

In the 2016 revision to the World Health Organization classification of myeloid neoplasms and acute leukemia [132], MPAL is categorized as MPAL, B/myeloid, not otherwise specified (NOS); MPAL, T/myeloid, NOS; and MPAL, NOS, rare types (T/B/myeloid). The disease has two genomic categories: (1) MPAL with t(9;22) (q34.1;q11.2); BCR-ABL1 and (2) MPAL with t(v;11q23.3); KMT2A-rearranged [132,133,134].

*ZNF384* rearrangement occurs in 40–50% of pediatric B/myeloid MPAL, but is rare in adult MPAL [133,134]. B/myeloid MPAL with *ZNF384* rearrangement and *KMT2A* rearrangement displays enhanced FLT3-mediated signaling regardless of whether somatic FLT3 mutations are present [134]. One study reported that FLT3-ITD is a recurrent mutation in MPAL and suggested that the immunophenotype and, hence, leukemogenesis differs between B/myeloid and T/myeloid MPAL [135]. Biallelic WT1 alterations are more frequent in T/myeloid MPAL, which has some genomic features similar to those of ETP-ALL [125].

Multinational retrospective studies have shown that an ALL-based induction regimen is more efficacious than an AML-like or combined-type regimen in patients with MPAL [133,136]. However, treatment can be switched to an AML-like regimen if induction therapy with an ALL-like regimen fails. Although the role of HSCT in the treatment of MPAL is controversial, there is a growing inclination towards using the modality after the first complete remission [134].

More recent clinical studies have shown that CD19 bispecific T-cell engagers and CAR T cells are promising treatments for MPAL [34,35] and that immunotherapy with blinatumomab can be used as a bridge to HSCT [36,37]. Another recent report described the successful treatment of a case of refractory, *KMT2A*-rearranged infant MPAL using a combination of immunotherapy agents targeting CD19 (blinatumomab) and CD33 (gemtuzumab) [137]. Additional studies are required to explore more therapeutic options.

## 3. Conclusions

Within these groups of refractory disease, the mechanism of therapy escape varies dramatically, making it highly important to identify markers of high-risk disease earlier in the diagnostic process. The burgeoning field of targeted therapy provides opportunities to incorporate novel effective agents into existing treatment backbones. From the first recognition of treatment-susceptible fusions in the Philadelphia chromosome to the recent implementation of menin inhibitors, the repertoire of ever-more-effective treatments for high-risk ALL continues to grow, comprising an increasing list of effective anti-leukemia agents: JAK-STAT and proteasome blockers that directly impact cancer biology, molecular markers for initiating the delivery of disease-specific chemotherapy, and immune-modulating and -activating agents. There is a continuous need to introduce these novel agents into existing leukemia regimens and investigate their effectiveness as both upfront therapy and, in the setting of relapse, salvage therapy. However, more agents and longer regimens do not always result in increased survival, and they are often associated with more acute or long-term sequelae. Thus, future studies must determine how these medications can be most efficaciously and efficiently evaluated and delivered to targeted populations. We must develop a paradigm in which such drugs are not only investigated as monotherapy, but also swiftly integrated into existing chemotherapy trials. In such a setting, we can continue to reduce high-risk ALL patients’ exposure to traditional chemotherapy—perhaps one day even eliminate it altogether—while continuing to accelerate improvements in their outcomes.

## Figures and Tables

**Table 1:** Survival outcomes and targeted therapies for patients with high-risk acute lymphoblastic leukemia.

ALL Type	5-Year EFS Rate	5-Year OS Rate	Targeted Therapies
Ph+	58 ± 6% (children and adolescents 1–21 years) [13]	70 ± 6% (children and adolescents 1–21 years) [13]	TKIs (imatinib, dasatinib) [13]
Ph-like	58.2 ± 5.3% (children 1–15 years), 41.0 ± 7.4% (adolescents 16–20 years), and 24.1 ± 10.5% (young adults 21–39 years) [14]	72.8 ± 4.8% (children 1–15 years), 65.8 ± 7.1% (adolescents 16–20 years), and 25.8 ± 9.9% (young adults 21–39 years) [14]	TKIs, JAK inhibitors (ruxolitinib) [15,16]
*KMT2A* rearranged	6-year EFS 73.9% (infants) [17]	6-year OS 87.2% (infants) [17]	Blinatumomab, CAR T-cells [18], menin inhibitors [19]
*MEF2D* rearranged	74% (63–82%) (children and adolescents 1–18 years) [20]	81% (71–88%) (children and adolescents 1–18 years) [20]	HDAC inhibitors [21], proteasome inhibitors [22]
*TCF3::HLF* fusion	25 ± 21.7% (infants, children, and adolescents 0–18 years) [23]	37.5 ± 28.6% (infants, children, and adolescents 0–18 years) [23]	BCL2 inhibitors [24], PARP inhibitors [25], blinatumomab followed by HSCT [26]
Hypodiploid	55.1% (95% CI, 49.3–61.5%) (infants, children, and adolescents up to 21 years) [27]	61.2% (95 CI, 55.5–67.4%) (infants, children, and adolescents up to 21 years [27]	BCL2 inhibitors, PI3K inhibitors, immunotherapy, CAR T-cells [22,28,29,30]
iAMP21	4-year EFS 72.7 ± 5.8% (children, adolescents, and young adults 1–30 years) [31]	4-year OS 87.6 ± 4.4% (children, adolescents, and young adults 1–30 years) [31]	Trials of target therapies are needed
ETP-ALL	87.0% (children, adolescents, and young adults 1–31 years) [32]	93.0% (children, adolescents, and young adults 1–31 years) [32]	Trials of target therapies are needed
MPAL	72 ± 8% (entire cohort 1–30 years), 75 ± 13% (patients on ALL regimen), 62 ± 14% (patients on AML regimen) [33]	77 ± 7% (entire cohort 1–30 years), 84 ± 9% (patients on ALL regimen), 69 ± 14% (patients on AML regimen) [33]	CD19 bispecific T-cell engagers and CAR T cells [34,35], immunotherapy with blinatumomab bridge to HSCT [36,37]

Abbreviations: EFS, event-free survival; OS, overall survival; Ph+ ALL, Philadelphia chromosome-positive ALL; TKI: tyrosine kinase inhibitor; Ph-like ALL, Philadelphia chromosome-like ALL; CAR, chimeric antigen receptor; HDAC, histone deacetylase; BCL2, B-cell lymphoma 2; PARP, poly(ADP-ribose) polymerase; CI, confidence interval; PI3K, phosphoinositide 3-kinase; iAMP21, intrachromosomal amplification of chromosome 21; ETP-ALL, early T-cell precursor ALL; MPAL, mixed-phenotype acute leukemia; HSCT, hematopoietic stem cell transplantation.

## Data Availability

No new data were created or analyzed in this study. Data sharing is not applicable to this article.
